# Cardiovascular magnetic resonance imaging characteristics in patients with methamphetamine-associated cardiomyopathy

**DOI:** 10.1186/s12968-022-00898-6

**Published:** 2022-12-01

**Authors:** Michael B. Stokes, Fiona Thoi, Daniel J. Scherer, Kyi T. H. Win, David M. Kaye, Karen S. Teo, Prashanthan Sanders

**Affiliations:** 1 School of Medicine, University of Adelaide, Adelaide, Australia; 2 Department of Cardiology, Central Adelaide Local Health Network, Adelaide, Australia; 3 Heart Health Theme, South Australian Health and Medical Research Institute, Adelaide, Australia; 4 Centre for Heart Rhythm Disorders, University of Adelaide, Adelaide, Australia; 5 Department of Clinical Research, The Baker Heart and Diabetes Institute, Melbourne, Australia; 6 Department of Cardiology, The Alfred Hospital, Melbourne, Australia; 7 Department of Medicine, Monash University, Clayton, VIC Australia

**Keywords:** Dilated cardiomyopathy, Heart failure, Imaging, Methamphetamine

## Abstract

**Background:**

Methamphetamine-associated cardiomyopathy (MA-CMP) is an increasingly recognised aetiology of cardiomyopathy. Cardiovascular magnetic resonance (CMR) is a specialised cardiac imaging modality commonly used in assessment of cardiomyopathy. We aimed to identify specific CMR features associated with MA-CMP.

**Methods:**

A retrospective cohort study of CMR scans was performed in a single centre between January 2015 and December 2020. Thirty patients with MA-CMP who had undergone CMR were identified. MA-CMP was defined as those with a history of significant methamphetamine use hospitalised with acute decompensated heart failure (other causes of cardiomyopathy excluded). A retrospective analysis of index admission CMRs was performed. All studies were performed on a 1.5 T CMR scanner.

**Results:**

The mean age of MA-CMP patients was 43.7 ± 7.5 years, and 86.7% were male. The mean left ventricular (LV) volume obtained in this cohort was consistent with severe LV dilatation (LV end-diastolic volume (334 ± 99 ml); LV end-systolic volume: 269 ± 98 ml), whilst the right ventricular (RV) volume indicated moderate-to-severe dilatation (RV end-diastolic volume: 272 ± 91 ml; RV end-systolic volume: 173 ± 82 ml). Mean LV ejection fraction (20.9 ± 9.2%) indicated severe LV dysfunction, with moderate-to-severe RV dysfunction also detected (RV ejection fraction: 29.4 ± 13.4%). 22 patients (73.3%) had myocardial late gadolinium enhancement (LGE), of which 59.1% were located in the mid-wall, with all of these involving the interventricular septum. 22.7% displayed localised regions of sub-endocardial LGE in a variety of locations, and 18.2% had transmural regions of LGE that were located in the inferior and inferolateral segments. 6 patients (20%) had intracardiac thrombus (4 LV, 2 both LV and RV).

**Conclusion:**

MA-CMP was associated with severe biventricular dilatation and dysfunction, with a high prevalence of intraventricular thrombus. This cohort study highlights that MA-CMP patients have a high prevalence of CMR findings.

## Background

The growth in methamphetamine usage worldwide continues to present increasing societal and healthcare challenges. With the escalation of its usage in a variety of social demographics, methamphetamine-associated cardiomyopathy (MA-CMP) has become an increasingly recognised aetiology of cardiomyopathy, particularly amongst younger patients presenting to emergency departments with heart failure (HF) symptoms and are found to have newly detected ventricular dysfunction [1–4]. The consequence of this disease process is development of HF with reduced ejection fraction (HFrEF) from severe ventricular dilatation and left ventricular (LV) dysfunction [5]. This clinical entity has substantially increased in its recognition and incidence over the past 15 years with a substantial increase in hospitalisations [6].

Although there has been a number of observational studies of this cohort [4, 5, 7–10], the complex aetiology of MA-CMP remains unclear, and the cardiovascular magnetic resonance (CMR) findings have yet to be characterised. CMR is a specialised cardiac imaging modality commonly used in assessment of cardiomyopathy with the potential for accurate biventricular volume measurement, calculation of ejection fraction, tissue characterisation and detection of intracardiac thrombus. We aimed to characterise the clinical and CMR features associated with MA-CMP. This is of particular importance as a significant burden of MA-CMP may be reversible with early intervention and complete drug abstinence [11–13]

## Methods

### Study population

A retrospective cohort study was performed of a population of patients with MA-CMP. Patients were identified at the Central Adelaide Local Health Network (CALHN, in Adelaide, South Australia), via a search of the HF database and searching through discharge summaries, utilising electronic medical records from the period between January 2015 and December 2020, to identify all patients with MA-CMP. MA-CMP was defined as those with a history of regular, long-term methamphetamine use, who had required inpatient cardiology admission with acute decompensated HF and had no other identifiable causes of cardiomyopathy. Specifically, coronary artery disease, primary valvular disease (either native or prosthetic valves), active hyperthyroidism, idiopathic, viral, alcohol, chemotherapy or hypertension-related cardiomyopathy were excluded where identified. If there was a history of alcohol or other significant drug use, it was assessed whether methamphetamine was the primary drug of dependence for participant inclusion. Patients were also excluded if they had no available CMR imaging, including those that could not tolerate CMR due to contraindications and/or claustrophobia. All studies were performed on a 1.5 T CMR scanner (Siemens Healthineers, Erlangen, Germany) with Gadovist (Bayer Healthcare, Berlin, Germany), the administered intravenous contrast agent.

### Data collection

Clinical and demographic data collected included age at time of index admission/diagnosis, sex, ethnicity, known route of methamphetamine ingestion, duration and/or cessation of methamphetamine use, use of tobacco products, excessive alcohol consumption, and other drug abuse, as well as medical comorbidities (hypertension, diabetes mellitus, hyperlipidaemia, atrial fibrillation/flutter, chronic kidney disease or ischaemic heart disease). Therapies patients were receiving for MA-CMP were also recorded along with patient compliance. CMR parameters collected included: LV end-diastolic volume (LVEDV), LV end-systolic volume (LVESV), LV ejection fraction (LVEF), right ventricular (RV) end-diastolic volume (RVEDV), RV end-systolic volume (RVESV), RV ejection fraction (RVEF, %), presence and location of late gadolinium enhancement (LGE) and presence of intra-cardiac thrombus, according to published standards [14].

### Statistical analysis

All continuous variables were presented as mean ± SD as appropriate to distribution. Categorical variables were expressed as number and percentage. Outcomes which did not follow the normal distribution, as assessed via plotting, were expressed as mean and range, with means tested using Mann–Whitney U. For all tests, significance was accepted as a p value < 0.05. All statistical analyses were conducted using SPSS (version 27, Statistical Package for the Social Sciences, International Business Machines, Inc., Armonk, New York, USA). The CALHN Human Research Ethics Committee of the Royal Adelaide Hospital approved the study protocol and waived informed consent.

## Results

**Fig. 1:**
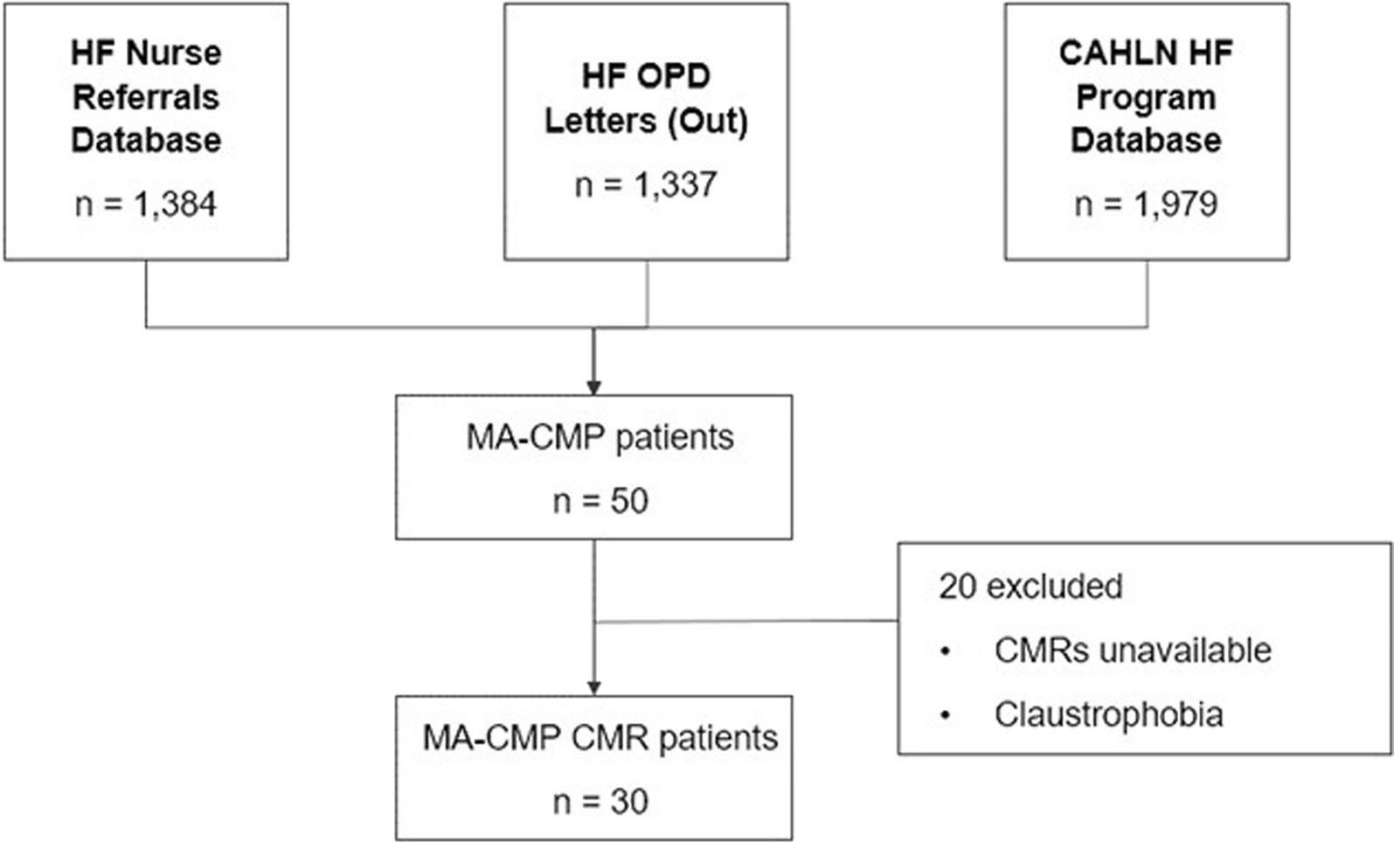
Study flowchart. Cohort selection diagram for the study cohort. *CALHN* Central Adelaide Local Health Network; *CMR* cardiovascular magnetic resonance; *HF* heart failure; *MA-CMP* methamphetamine-associated cardiomyopathy; *OPD* outpatient department

A total of 4,700 consecutive patients with a history of HF were identified during the study period. After exclusion criteria were applied, 30 patients were included in the cohort. Cohort selection flowchart is shown in Fig. 1.

**Fig. 2:**
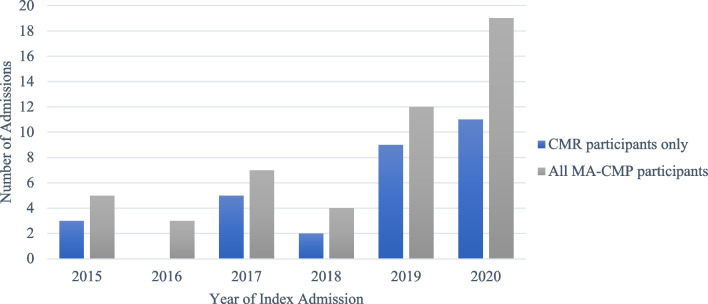
Incidence of MA-CMP across the years assessed in this cohort (30 participants), as well as the overall 50 subjects with MA-CMP (including those that did not have a CMR)

The year of diagnosis was recorded with an increase in incidence evident across the data collection period, both within the population with CMR imaging performed, and those without, as seen in Fig. 2.

Table 1 outlines the baseline information for this cohort. Most MA-CMP patients were in their fifth decade of life and predominantly male. The average duration of methamphetamine use was 13.4 years, with a comparable portion who were chronic intravenous users (43.3%); whilst others smoked (53.3%), inhaled (6.7%) or had a mixture of consumption methods (13.3%).

**Table 1:** Participant demographic & clinical characteristics

	Participants (n = 30)
Age	43.7 ± 7.5
Male	26 (86.7)
Methamphetamine use	
Duration of use	13.4 ± 4.9
Intravenous users	13 (43.3)
History of substance abuse	
Smoking	24 (80)
Alcohol	12 (40)
Marijuana	15 (50)
Comorbidities	
Hypertension	10 (33.3)
Diabetes mellitus	3 (10)
Hyperlipidaemia	3 (10)
Atrial fibrillation/flutter	0
Chronic kidney disease	1 (3.3)
Prior ischaemic heart disease	1 (3.3)

Data are mean ± SD or n (%)

Many MA-CMP patients had concurrent heavy tobacco use (80%), and a portion had a previous or current history of excess alcohol use (40%) and/or marijuana use (50%). Comorbidities were present within the cohort, with overall 33.3% possessing a history of hypertension, 10% diabetes mellitus, 10% hyperlipidaemia, 3.3% chronic kidney disease and 3.3% with prior ischaemic heart disease. No patients had a history of cardiac rhythm disorders.

CMR parameters are presented in Table 2. The mean LV volume obtained of the participants included was consistent with severe LV dilatation (LVEDV 334 ± 99 ml; LVESV: 269 ± 98 ml), whilst the mean RV volume indicated moderate-to-severe RV dilatation (RVEDV: 272 ± 91 ml; RVESV: 173 ± 82 ml). LVEF (20.9 ± 9.2%) indicated severe LV dysfunction, with moderate-to-severe RV dysfunction also detected (RVEF: 29.4 ± 13.4%) in all MA-CMP patients.

**Table 2:** Study cohort CMR parameters

Variable	Participants	Normal range (Ref. [14])
LVEDV, ml	334 ± 99	109–218
LVEDVI, ml/m^2^	157 ± 37	
LVESV, ml	269 ± 98	39–97
LVESVI, ml/m^2^	126± 41	
LVEF, %	20.9 ± 9.2	48–69
RVEDV, ml	272 ± 91	124–248
RVEDVI, ml/m^2^	121 ± 33	
RVESV, ml	173 ± 82	47–123
RVESVI, ml/m^2^	80 ± 34	
RVEF, %	29.4 ± 13.4	45–65

Data are mean ± SD

*LVEDV* left ventricular end diastolic volume; *LVEDVI* left ventricular end diastolic volume index; *LVESV* left ventricular end systolic volume; *LVESVI* left ventricular end systolic volume index; *LVEF* left ventricular ejection fraction; *RVEDV* right ventricular end diastolic volume; *RVEDVI* right ventricular end diastolic volume index; *RVESV* right ventricular end systolic volume; *RVESVI* right ventricular end systolic volume index; *RVEF* right ventricular ejection fraction

**Fig. 3:**
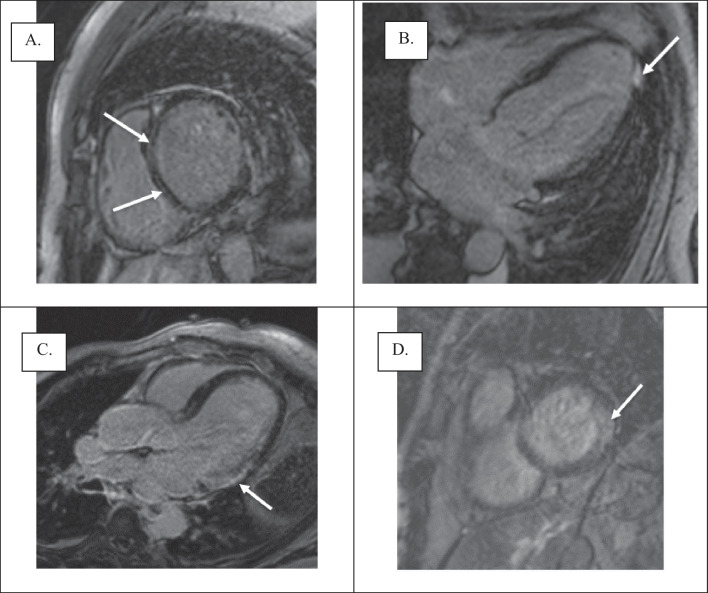
Presence and location of LGE (3 studies). **A** Study 1: Mid wall linear LGE (septal); short axis image. **B** Study 2: Focal transmural LGE at apical inferolateral segment; 4 chamber image. **C** Study 3: Subendocardial LGE at basal infero-lateral segment; 3 chambers view. **D** Study 3: Transmural LGE at basal infero-lateral segment; short axis image

Table 3 demonstrates the findings related to presence and location of LGE on CMR. Twenty-two patients (73.3%) had myocardial LGE, of which 59.1% were located in the mid-wall, with all of these involving the interventricular septum. 22.7% displayed localised regions of sub-endocardial LGE in a variety of locations and 18.2% had transmural regions of LGE that were located in the inferior and inferolateral segments. Several case studies are shown in Fig. 3 to highlight these features.

**Table 3:** Presence and location of LGE data

Myocardial LGE	Participants (n = 22; 73.3%)
Mid-wall (including septal involvement)	13 (59.1)
Sub-endocardial	5 (22.7)
Transmural	4 (18.2)

Data are n (%)

*LGE* late gadolinium enhancement

**Fig. 4:**
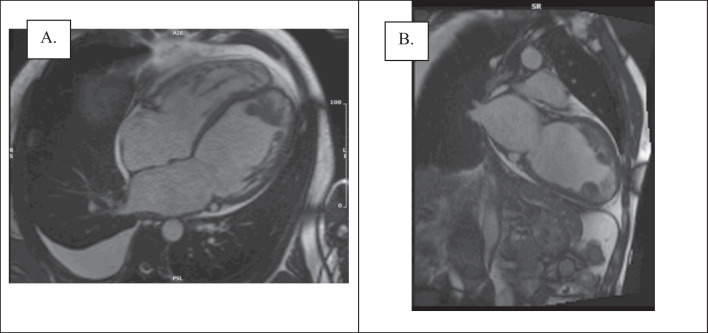
Visualisation of left ventricular thrombi burden identified on CMR (same study). **A** 4-chamber view; **B** 2-chamber view

Six patients (20%) had intracardiac thrombus. Of these cases, 66.7% were in the LV and 33.3% were biventricular. Figure 4 demonstrates one of the cases in which the burden was limited to the LV. Multiple, irregular large LV thrombi appeared on the horizontal long-axis view at the apex and were circular appearing at the apex on the vertical long-axis view. They had an irregular appearance involving the basal-to-mid anterior segments, with some tracking along the mid anterior segment.

Follow-up data were available on 24 of the 30 patients (80%) included in this cohort using echocardiography. Complete cessation of methamphetamine use and compliance to HF pharmacotherapy was advised to all included participants in this cohort during their index admission. Of the cohort of 11 patients who ceased methamphetamine, all (100%) complied with their medication regime. This compared with only 5 out of the 13 patients taking prescribed medications who continued to use methamphetamine following diagnosis. Over a median follow-up of 6 months (IQR 3.0 to 6.5 months) from index admission, improvement in cardiac function, specifically a LVEF increase of > 5%, was observed in participants who achieved complete cessation of their methamphetamine use compared to those who reduced or remained consistent in their consumption (p-value = 0.003). The presence of baseline LGE on CMR, however, did not show a significant impact on change in follow up LVEF. Additionally, whilst there were variable cessation rates in both patients with and without LGE, a two-way ANOVA test indicates that only cessation is statistically significant (p-value = 0.009), independent of LGE. These figures are presented in Tables 4, 5 and 6.

**Table 4:** Follow-up left ventricular ejection fraction (LVEF) data for 24 MA-CMP patients following treatment advice

	Metamphetamine cessation (n = 11)	Continued metamphetamine use (n = 13)	p-value
Change in EF, %	16 (5 to 22)	3.1 (− 12 to 34)	0.003

Data are mean (range)

**Table 5:** Follow-up LVEF data for 24 MA-CMP patients comparing the presence of LGE on index CMR

	Baseline LGE (n = 17)	No Baseline LGE (n = 7)	p-value
Change in LVEF, %	11.1 (− 11 to 34)	3.6 (− 12 to 12)	0.135
Cessation	47.1	42.8	

Data are mean (range) or n (%)

**Table 6:** Results of the two-way ANOVA to analyse the effect of LGE and Cessation on follow-up LVEF

Source of variation	F	p-value
LGE	2.250	0.149
Cessation	8.233	0.009
Interaction	0.004	0.951

**Fig. 5:**
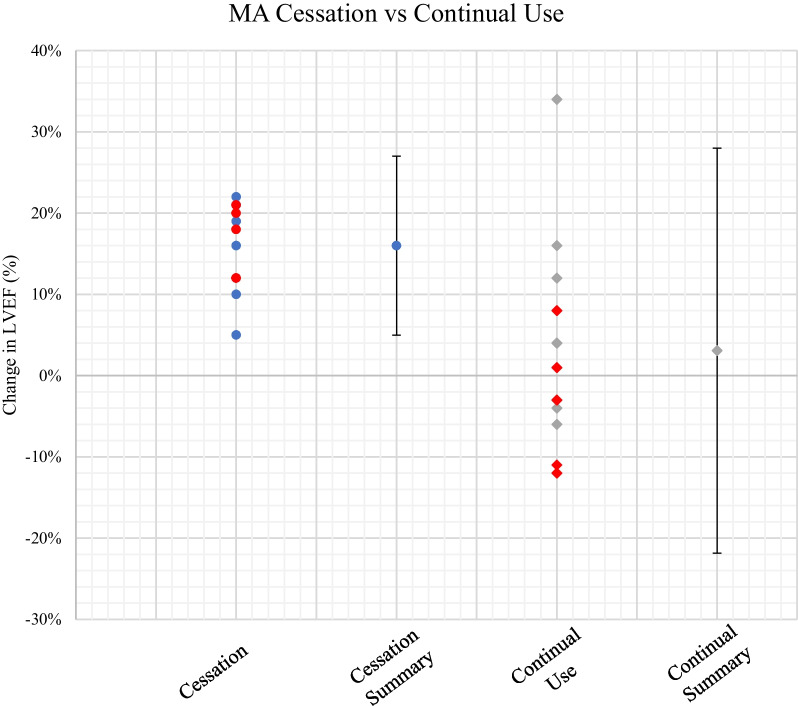
Follow up LVEF data for 24 MA-CMP patients comparing the effect of methamphetamine cessation (circles) with continual use (diamonds), with participants with a history of alcohol abuse further visualised in red in both groups. Mean data ± 2 standard deviations for both groups are also plotted for further comparison

Given the potential of alcohol abuse being a confounder and contributor to cardiomyopathy in this population, the relationship of alcohol abuse on ventricular volumes, LVEF and RVEF (at baseline), as well as recovery in ventricular function was measured. Whilst there was a small trend to increased volumes in those with a history of alcohol abuse (Table 7), this was not significant. In addition, Fig. 5 highlights that a history of prior alcohol use did not affect rates of improvement in ventricular function with methamphetamine cessation, as patients who ceased methamphetamine use did not also cease alcohol use.

**Table 7:** Study cohort CMR parameters comparing participants with a history of alcohol abuse versus those without

Variable	Alcohol history (n = 13)	Nil alcohol history (n = 17)	p-value
LVEDV, ml	360 ± 115	314 ± 86	0.230
LVESV, ml	258 ± 112	247 ± 88	0.206
LVEF, %	18.2 ± 7.9	22.4 ± 10.3	0.293
RVEDV, ml	257 ± 111	225 ± 70	0.350
RVESV, ml	194 ± 97	164 ± 71	0.277
RVEF, %	27.2 ± 12.4	28.5 ± 13.5	0.412

Data are mean ± SD

## Discussion

CMR has evolved as an essential investigative tool in evaluation of cardiomyopathy due to its accurate quantification of biventricular volumes and ejection fraction as well as tissue characterisation [15]. In addition, detection of specific patterns of LGE and assessment of its burden does assist in assessment of risk for arrhythmias and prognosis in non-ischaemic cardiomyopathy. In this retrospective study of an important HF group, we identified a number of adverse prognostic markers on CMR in patients with MA-CMP.

LV dilation and contractile impairment is a significant predictor of adverse outcomes in dilated cardiomyopathy [16]. In addition, RV dilatation and left atrial dilatation also confer significant prognostic value in cardiomyopathy [17, 18]. Our retrospective study has demonstrated a substantial degree of LV and RV dilatation as measured on CMR. A follow-up CMR study assessing the effects of medical therapy and prognosis relating to degree of ventricular dilatation would provide further insights into prognosis of MA-CMP.

The mechanisms of ventricular dilatation specifically in MA-CMP warrant discussion. MA-CMP development is postulated to be a secondary to an interplay of direct and indirect mechanistic pathways. Directly, amphetamines promote free radical production resulting in direct cellular injury, apoptosis via increased p53 activity, mitochondrial dysfunction, altered gene expression and defective intracellular haemostasis. Contractile dysfunction develops from disrupted electrical–mechanical coupling [19]. In susceptible individuals, long-term use results low-grade inflammation and consequent myocyte loss with replacement fibrosis. Indirectly, amphetamine does potentiate heightened sympathetic nervous system activity, which can also induce coronary and microvascular spasm, resulting in acute ischaemia [20–22]. These mechanisms consequently may result in the development of significantly dilated cardiac chambers, systolic dysfunction, and clinical presentation of the heart failure syndrome [5, 23, 24].

LGE is an effective, well-validated and reproducible method of detecting myocardial fibrosis and other pathologies such as infarction, infiltration and inflammation. An abundance of CMR clinical studies have demonstrated correlation with adverse events (including sudden cardiac death in non-ischaemic cardiomyopathy [25–27]. The incidence of LGE in our population of MA-CMP was quite high (76.3%), and the pattern of LGE identified in our cohort was heterogenous. This implies varied pathological processes that may occur with MA-CMP including chronic stress precipitating mid-wall fibrosis, micro-embolic phenomena and possibly areas of low-grade chronic inflammation. The majority of patients (56.5%) had evidence of mid-wall LGE, indicating that chronic inflammation and resultant mid-wall fibrosis is likely to be the predominant mechanism for developing of cardiomyopathy. However, 21.7% of the patients included had evidence of sub-endocardial LGE and 21.7% had transmural LGE, both of which can typically be indicative of prior myocardial infarction. In the population included, all patients had obstructive coronary artery disease excluded at coronary angiography which supports the hypothesis of these patients having possible coronary arterial spasm or myocardial infarction with non-obstructive coronary arteries (MINOCA) as the aetiology. The presence and burden of LGE detected on CMR in the non-ischaemic population has been demonstrated to be a powerful imaging biomarker in predicting adverse outcomes and response to medical therapy [28]. In addition, a previous study of 30 patients undergoing endomyocardial biopsy in MA-CMP has reported the presence of fibrosis on biopsy representing an adverse relationship with recovery of ventricular function [5]. Therefore, in the population of MA-CMP where individual clinical factors may render some patients challenging for device implantation, the further prognostic information from LGE assessment may assist in important clinical decision making with regard to implanted cardiodefibrillator (ICD) placement and may also assist with pharmacotherapy selection as well as discussions with patients regarding long-term prognosis.

The incidence of ventricular thrombi was also a highly relevant clinical finding in the population studied. Intra-cardiac thrombi carry significant clinical risk with embolization and its detection has a profound impact in management by mandating therapeutic anticoagulation [29]. Intra-cardiac thrombi have been observed in other studies assessing the MA-CMP population [5]. Our study revealed a 20% incidence of ventricular thrombus with MA-CMP, observed in mixed patterns of LGE. Given the potential clinical catastrophic consequences, this finding necessitates an adequately thorough exclusion for ventricular thrombus with patients with MA-CMP that undergo cardiac imaging.

CMR sequences that were not available in our cohort but would be of value in a future study include native T1 mapping, post contrast T1 mapping and patterns in tissue tracking measuring LV, RV and left atrial strain. Data from these sequences would provide further mechanistic and prognostic insights into the entity of MA-CMP, specifically around the incidence of diffuse fibrosis, long-axis LV function and left atrial function.

Dedicated left atrial volume acquisition may also have provided additional data in this patient population given the prognostic value of atrial dilatation in cardiomyopathy.

### Study limitations

Limitations of our study include the obligatory weaknesses associated with a retrospective observational design performed via reviewing medical records which may have resulted in a degree of selection bias. In addition, there was a lack of a control group of non-ischaemic cardiomyopathy which was chosen not to be included due to the heterogeneity of various aetiologies of non-ischaemic cardiomyopathy. Genetic testing for familial dilated cardiomyopathy was also not available in our cohort, however, there was no identified familial cardiomyopathy included in the studied population. Data was gathered from a single healthcare network and may not be as applicable to other populations. However, this limitation is offset by the advantages of this single tertiary centre including uniformity in clinical practice, data acquisition and consistency in CMR study interpretation.

A significant cohort of the population included also had a history of alcohol abuse (~ 40%). However, patients included were assessed on review of the medical records as to what was their predominant drug of abuse and dependence. Patients only with predominant MA abuse were included in our study, but alcohol certainly may have been a contributor to development of a cardiomyopathy in those with a history of alcohol abuse which does represent a potential confounder to the findings reported. Importantly, there was no statistically significant difference in the baseline ventricular volumes in those with previous alcohol abuse, compared to those who did not.

The population selected for this cohort had a significant and long-history of methamphetamine abuse. However, the intensity of methamphetamine use is difficult to quantify in this patient population, particularly when clinical data is retrieved retrospectively from reviewing electronic medical records. Therefore, the study does represent a reasonably heterogenous population of methamphetamine users, with variable methods and levels of consumption.

## Conclusions

To our knowledge, this is the largest series of MA-CMP that has been characterised with CMR. This study demonstrated that MA-CMP is associated with significant biventricular dilatation and systolic dysfunction, a high incidence of LGE and a 20% incidence of intra-cardiac thrombus, which was sometimes biventricular. The MA-CMP population possessed heterogenous patterns of LGE detected, most frequently observed in the mid-wall and interventricular septum. Follow-up data of this cohort revealed that abstinence from methamphetamine use following diagnosis of MA-CMP was associated with a greater improvement in LVEF. Future studies with larger cohorts, as well as outpatient cohorts, are required to corroborate these findings and to investigate their follow-up imaging for further recovery of cardiac function.

## Data Availability

The datasets used and/or analysed during the current study are available from the corresponding author on reasonable request.

## Abbreviations

CMR: Cardiovascular magnetic resonance
HF: Heart failure
LGE: Late gadolinium enhancement
LV: Left ventricle/left ventricular
LVEDV: Left ventricular end-diastolic volume
LVEDVI: Left ventricular end-diastolic volume index
LVEF: Left ventricular ejection fraction
LVESV: Left ventricular end-systolic volume
LVESVI: Left ventricular end-systolic volume index
MA-CMP: Methamphetamine-associated cardiomyopathy
RV: Right ventricle/right ventricular
RVEDV: Right ventricular end-diastolic volume
RVEDVI: Right ventricular end-diastolic volume index
RVEF: Right ventricular ejection fraction
RVESV: Right ventricular end-systolic volume
RVESVI: Right ventricular end-systolic volume index
